# The properties of Msh2–Msh6 ATP binding mutants suggest a signal amplification mechanism in DNA mismatch repair

**DOI:** 10.1074/jbc.RA118.005439

**Published:** 2018-09-20

**Authors:** William J. Graham, Christopher D. Putnam, Richard D. Kolodner

**Affiliations:** From the ‡ Ludwig Institute for Cancer Research San Diego,; Departments of § Medicine and; ¶ Cellular and Molecular Medicine,; ‖ Moores-UCSD Cancer Center, and; ** Institute of Genomic Medicine, University of California School of Medicine, San Diego, La Jolla, California 92093-0669

**Keywords:** DNA repair, DNA replication, DNA mismatch repair, DNA endonuclease, DNA binding protein, mutagenesis, exonuclease 1, Mlh1-Pms1, Msh2-Msh6, MutS, S. cerevisiae

## Abstract

DNA mismatch repair (MMR) corrects mispaired DNA bases and small insertion/deletion loops generated by DNA replication errors. After binding a mispair, the eukaryotic mispair recognition complex Msh2–Msh6 binds ATP in both of its nucleotide-binding sites, which induces a conformational change resulting in the formation of an Msh2–Msh6 sliding clamp that releases from the mispair and slides freely along the DNA. However, the roles that Msh2–Msh6 sliding clamps play in MMR remain poorly understood. Here, using *Saccharomyces cerevisiae,* we created Msh2 and Msh6 Walker A nucleotide–binding site mutants that have defects in ATP binding in one or both nucleotide-binding sites of the Msh2–Msh6 heterodimer. We found that these mutations cause a complete MMR defect *in vivo*. The mutant Msh2–Msh6 complexes exhibited normal mispair recognition and were proficient at recruiting the MMR endonuclease Mlh1–Pms1 to mispaired DNA. At physiological (2.5 mm) ATP concentration, the mutant complexes displayed modest partial defects in supporting MMR in reconstituted Mlh1–Pms1-independent and Mlh1–Pms1-dependent MMR reactions *in vitro* and in activation of the Mlh1–Pms1 endonuclease and showed a more severe defect at low (0.1 mm) ATP concentration. In contrast, five of the mutants were completely defective and one was mostly defective for sliding clamp formation at high and low ATP concentrations. These findings suggest that mispair-dependent sliding clamp formation triggers binding of additional Msh2–Msh6 complexes and that further recruitment of additional downstream MMR proteins is required for signal amplification of mispair binding during MMR.

## Introduction

DNA mismatch repair (MMR)^2^ is a conserved pathway that repairs mispaired bases that result from errors during DNA synthesis (1–6). MMR also acts on some forms of chemically damaged DNA bases as well as on mispairs present in heteroduplex DNA intermediates formed during recombination (1–6). MMR proteins also act in a pathway that suppresses recombination between homologous but divergent DNAs (1–6) and function in a DNA damage response pathway for different types of DNA-damaging agents, including some chemotherapeutic agents (2). As a result of the role that MMR plays in correcting mispairs, mutations in or loss of expression of MMR genes results in increased mutation rates that underlie both sporadic cancers and inherited cancer predisposition syndromes in humans (7–11).

The initiation of MMR in eukaryotes relies upon the recognition of DNA mispairs by the partially redundant Msh2–Msh6 and Msh2–Msh3 heterodimers, which are homologs of the bacterial MutS homodimer (1, 3, 12). Msh2–Msh6, Msh2–Msh3, and MutS belong to the ABC family of ATPases and have two composite nucleotide-binding sites at their dimeric interface (13–17). Each composite nucleotide-binding site comprises the Walker A and Walker B motifs from one subunit and the “signature” motif from the other subunit (18–20). In the MSH complexes, of which Msh2–Msh6 has been the most extensively studied, ATP binding, ATP hydrolysis, and ADP release are intrinsically linked to conformational changes that play critical roles in MMR (20–34). In the Msh2–Msh6 complex, the Msh6 subunit, which directly interacts with the mispair, has the higher affinity for ATP (∼10-fold higher than Msh2), whereas the Msh2 subunit has the higher affinity for ADP (24). Similarly, the homodimeric bacterial MutS, which adopts an asymmetric conformation upon mispair recognition (13, 14), also has ATP binding asymmetries (25, 35, 36). In both human and *Saccharomyces cerevisiae* Msh2–Msh6, the Msh2 subunit controls the cycle of ATP processing. After Msh2–Msh6 binds mispaired DNA, release of Mg^2+^ from the Msh2 nucleotide-binding site causes release of ADP from Msh2, which then allows Msh6 to bind ATP (23, 24, 37). In turn, ATP binding to the Msh6 nucleotide-binding site results in reduced affinity of the Msh2 nucleotide-binding site for ADP, causing Msh2 to favor binding of ATP (24). Msh2–Msh6 binding to mispaired DNA also results in a reduction of the ATPase activity of Msh6 (26).

When Msh2–Msh6 and other members of the MutS family of MMR proteins bind to mispaired bases, they form a ring around the DNA with the mispair recognition domain of Msh6 (or Msh3 or the mispair-binding subunit of MutS) making contacts with both the mispair and adjacent sites on the DNA; these contacts also result in bending of the DNA (13, 14, 16, 17, 38, 39). ATP binding by mispair-bound Msh2–Msh6 and other MutS family members induces a number of conformational changes in the protein complex: 1) the DNA mispair recognition domain and the connector domain become exposed (29, 40), and 2) upon binding ATP in both ATP-binding sites, Msh2–Msh6 and other MutS family proteins transition into a sliding clamp form that dissociates from the mispair, alleviates the DNA bend at the mispair, and slides along the DNA (22–24, 31–33, 38, 41–43). In addition, mispair and ATP binding by Msh2–Msh6 results in recruitment of the MutL homolog complexes Mlh1–Pms1 and Mlh1–Mlh2 by the newly exposed surfaces on the connector domain of Msh2 (29, 40), which then appears to allow loading of Mlh1–Pms1 and Mlh1–Mlh2 onto the DNA and can mediate the formation of a MutL-based sliding clamp (31, 41, 43–45). However, studies of dominant *MSH6* mutant Msh2–Msh6 complexes have suggested that recruitment of Mlh1–Pms1 by *S. cerevisiae* Msh2–Msh6 may not be completely dependent on the formation of Msh2–Msh6 sliding clamps (21, 30).

Genetic studies of the Walker A and Walker B motifs have provided evidence that ATP binding and hydrolysis are essential for Msh2–Msh6 function during MMR. Walker A mutations that reduce ATP binding cause an MMR defect in *S. cerevisiae* (46, 47). These mutations did not affect the recognition of mispaired bases by Msh2–Msh6 in gel shift and filter binding assays but did eliminate the ability of added ATP to prevent the formation of Msh2–Msh6-mispaired DNA complexes (46, 47). Mutations that result in amino acid substitutions at the conserved Walker B Glu residue of *S. cerevisiae* Msh2 and Msh6 that cause ATP hydrolysis defects similarly resulted in MMR defects but did not affect either mispair recognition or ATP inhibition of formation of Msh2–Msh6-mispaired DNA complexes as detected in gel shift assays (48). Studies of human MSH2–MSH6 have yielded somewhat different results. Amino acid substitutions at the conserved Walker A Lys residue of either MSH2 or MSH6 resulted in mutant human MSH2–MSH6 complexes that recognized mispaired bases in gel shift assays, had ATP binding defects, showed partial resistance to ATP inhibition of mispair binding, and caused partial defects in the ability of MSH2–MSH6 to complement MMR-defective extracts of HCT15 cells in MMR reactions *in vitro* (49); the MSH2-Lys–MSH6-Lys double mutant protein had more striking defects in the latter two assays (49). A more recent study showed that Walker A Lys mutations affecting human MSH2 or MSH6 resulted in MSH2–MSH6 complexes that bound mispairs and exhibited ATP-induced dissociation and partially defective formation of sliding clamps (37). In contrast, double Walker A Lys mutations were completely defective in ATP-dependent processes (37). In addition, a Walker B Glu mutation in human *MSH6* was found to result in a mutant human MSH2–MSH6 complex that exhibited partial defects in an extract-based MMR reaction *in vitro* and a partial defect in ATP-dependent binding to an end-blocked mispaired DNA substrate (50). Complementary experiments analyzing dominant mutations that altered amino acids near the *S. cerevisiae* Msh6 or Msh2 nucleotide-binding sites but did not affect key catalytic amino acids have also provided evidence that ATP-induced conformational changes of Msh2–Msh6 are critical to MMR (21, 30, 51–54).

The studies performed to date have provided evidence that the Msh2–Msh6 ATP processing cycle is important for MMR. However, it is unclear how mutations affecting the Walker A and B motifs result in MMR defects. Here, we mutated the lysine codon (Lys^694^ in *S. cerevisiae* Msh2 and Lys^988^ in *S. cerevisiae* Msh6) in the Walker A motif to alanine, arginine, or methionine codons, resulting in six mutant complexes, each with one WT subunit and one mutant subunit. We examined the mutations and resulting mutant Msh2–Msh6 complexes using a battery of biochemical and genetic assays, many of which have never been used to characterize Msh2–Msh6 Walker A mutants. These mutations caused complete MMR defects *in vivo* and affected the ATP binding affinity of one or both of the Msh2 and Msh6 nucleotide-binding sites. Consistent with previous studies, none of these mutations altered mispair recognition. Provided that ATP concentrations were high enough to mitigate ATP binding defects of the Walker A Lys mutant complexes, all of the mutant complexes supported Mlh1–Pms1 recruitment to DNA, reconstituted MMR reactions, and activation of mispair-dependent Mlh1–Pms1 endonuclease activity to a large extent. In contrast, these mutant complexes had a profound defect in forming sliding clamps even at high ATP concentrations. Taken together, these data are consistent with the hypothesis that sliding clamp formation is required for MMR *in vivo*, most likely because mispair-dependent accumulation of multiple Msh2–Msh6 complexes per mispair is required for MMR.

## Results

### Msh2 and Msh6 Walker A mutant complexes have defects in ATP binding

**Figure 1.**
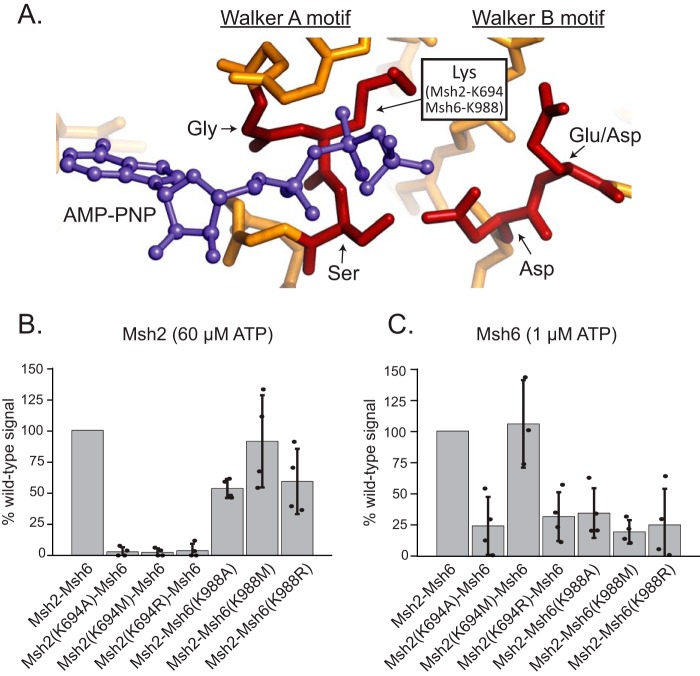
**Effects of mutations on the ability of mutant subunits to bind ATP.** *A*, ATP-binding site from *E. coli* MutS (Protein Data Bank (PDB) code 5akb (29)) depicting highly conserved residues from the Walker A motif (Lys^694^ in *S. cerevisiae* Msh2 and Lys^988^ in *S. cerevisiae* Msh6) and their position relative to an AMP-PNP ATP analog. *B*, histogram showing relative Msh2 binding of 60 μm [γ-^32^P]ATP under nonhydrolyzable conditions. *C*, histogram showing relative Msh6 binding of 1 μm [γ-^32^P]ATP under nonhydrolyzable conditions. *Error bars* represent the S.D. from the mean, and individual values from different experiments are indicated by the *dots* on each histogram *bar*.

When WT Msh2–Msh6 is bound to a mispair, ATP binding at both the high- (Msh6) and low (Msh2)-affinity nucleotide-binding sites triggers a conformational change from a relatively static mispair recognition complex to a clamp that can slide along the DNA (21–24, 32, 55). To study the role of ATP-induced conformation changes during *S. cerevisiae* MMR, we mutated the invariant lysine residue in the Walker A motif (Msh2-Lys^694^ and Msh6-Lys^988^; Fig. 1*A*) to alanine, methionine, or arginine, resulting in six mutant alleles: *msh2-K694A*, *msh2-K694M*, *msh2-K694R*, *msh6-K988A*, *msh6-K988M*, and *msh6-K988R*. This invariant lysine coordinates the γ-phosphate of bound ATP, and lysine to alanine or methionine amino acid substitutions in related ATPases often prevents ATP binding (24, 56), whereas lysine to arginine amino acid substitutions often, but not always, result in mutants that can bind, but not hydrolyze, ATP (56). For the sake of brevity, we refer to these six mutations collectively as Walker A Lys mutations/mutants.

To assess the ability of the mutant complexes to bind ATP, we utilized an assay where [γ-^32^P]ATP was bound to Msh2–Msh6 under conditions where hydrolysis was inhibited (2.5 mm EDTA and no Mg^2+^), ATP was cross-linked to the protein using UV irradiation, and samples were analyzed by SDS-PAGE followed by autoradiography. In the case of the heterodimeric Msh2–Msh6 complex, specific cross-linking to each subunit can be visualized as Msh2 and Msh6 have different molecular weights (21, 24, 57). The ATP concentrations used were chosen to be modestly above the previously determined *K_d_* values for Msh2 (∼40 μm) and Msh6 (∼0.3 μm) (24), and ATP binding at these concentrations was compared with WT Msh2–Msh6 (Fig. 1, *B* and *C*) (24). The Msh2(K694M)–Msh6 mutant complex was only defective for binding ATP to the Msh2 subunit, whereas the Msh2(K694A)–Msh6 and Msh2(K694R)–Msh6 mutant complexes were defective for ATP binding to both the Msh2 and Msh6 subunits (Fig. 1, *B* and *C*). The Msh6 subunits of each Msh6 mutant complex were defective for ATP binding at 1 μm ATP, whereas among the Msh6 mutant complexes the Msh2–Msh6(K988A) and Msh2–Msh6(K988R) mutant complexes had partial ATP binding defects at the Msh2 subunit at 60 μm ATP (Fig. 1, *B* and *C*). Thus, all of the six mutant complexes were defective for ATP binding to at least one subunit. We separated the six mutant complexes into two classes of ATP binding alterations: 1) ATP binding defects in both subunits (Msh2(K694A)–Msh6 and Msh2(K694R)–Msh6) and 2) ATP binding defects in the mutated subunit with partial or no effect on the other subunit (Msh2(K694M)–Msh6, Msh2–Msh6(K988A), Msh2–Msh6(K988M), and Msh2–Msh6(K988R)).

### The msh2 and msh6 Walker A motif mutations cause complete MMR defects in vivo

To determine whether the Walker A Lys mutations affect MMR *in vivo*, we integrated the mutations at the native *MSH2* or *MSH6* chromosomal loci as appropriate and tested their effects on mutation rates in the *hom3–10* and *lys2–10A* frameshift reversion rate assays and the *CAN1* forward mutation rate assay. The three Walker A Lys *msh2* mutations caused increased mutation rates in all three assays that were equivalent to those caused by an *msh2*Δ mutation, consistent with causing a complete MMR defect (Table 1). Similarly, the three Walker A Lys *msh6* mutations caused increases in mutation rates that were indistinguishable from the mutation rates caused by an *msh6*Δ mutation (Table 1). Msh2–Msh6 and Msh2–Msh3 are redundant in the repair of insertion/deletion mispairs (12), and consistent with this, combining the *msh6* Walker A Lys mutations with an *msh3*Δ mutation caused synergistically increased mutation rates in the *hom3–10* and *lys2–10A* frameshift reversion rate assays and a modest increase in the *CAN1* forward mutation rate assay, resulting in mutation rates that were equivalent to that caused by the *msh2*Δ single and *msh3*Δ *msh6*Δ double mutations (Table 1). Previous studies that examined *S. cerevisiae* Walker A *msh2* mutations on an *ARS CEN* plasmid found that these mutant alleles did not complement the mutator phenotype of an *msh2*Δ strain (46, 47). We conclude that the *MSH2* and *MSH6* Walker A Lys mutations are complete loss-of-function mutations *in vivo*.

**Table 1:** **Mutation rate analysis of msh2 and msh6 ATPase mutations** Median rates of *hom3–10* (Thr^+^) and *lys2–10A* (Lys^+^) reversion and inactivation of *CAN1* (Can^R^) are shown. The 95% confidence intervals are reported in brackets, and the -fold increases over wildtype are in parentheses.

Relevant genotype	RDKY no.	Thr^+^ mutation rate	Lys^+^ mutation rate	Can^R^ mutation rate
Wildtype	5964	1.52 [1.22–3.30] × 10^−9^ (1)	8.53 [6.67–18.6] × 10^−9^ (1)	3.87 [3.56–8.53] × 10^−8^ (1)
*msh2*Δ	3688	6.74 [5.00–15.9] × 10^−6^ (4,430)	8.04 [6.79–12.8] × 10^−5^ (9,430)	5.87 [3.97–8.30] × 10^−6^ (152)
*msh2-K694A*	9379	6.32 [5.64–10.8] × 10^−6^ (4,160)	7.96 [5.95–14.2] × 10^−5^ (9,330)	5.45 [4.42–7.81] × 10^−6^ (141)
*msh2-K694M*	9381	9.22 [6.35–11.3] × 10^−6^ (6,070)	1.30 [0.70–1.79] × 10^−4^ (15,200)	4.85 [3.92–6.16] × 10^−6^ (125)
*msh2-K694R*	9380	6.46 [4.94–10.8] × 10^−6^ (4,250)	9.50 [7.74–12.7] × 10^−5^ (11,100)	5.17 [3.78–5.96] × 10^−6^ (134)
*msh6*Δ	7965	2.61 [0.35–3.56] × 10^−8^ (17.2)	9.04 [3.75–12.7] × 10^−7^ (106)	1.36 [0.98–2.25] × 10^−6^ (35.1)
*msh6-K988A*	9382	2.99 [1.86–6.12] × 10^−8^ (19.7)	8.80 [6.79–12.5] × 10^−7^ (103)	1.30 [0.94–1.97] × 10^−6^ (33.6)
*msh6-K988M*	9384	1.57 [1.27–2.80] × 10^−8^ (10.3)	1.47 [0.94–2.17] × 10^−6^ (172)	1.23 [0.69–1.92] × 10^−6^ (31.8)
*msh6-K988R*	9383	2.52 [1.78–4.81] × 10^−8^ (16.5)	1.43 [1.07–1.94] × 10^−6^ (168)	1.17 [9.54–16.3] × 10^−6^ (30.2)
*msh3*Δ*^a^*	4149	2.2 [1.6–3.5] × 10^−8^ (14.5)	1.3 [0.87–1.9] × 10^−7^ (15.2)	1.1 [0.5–1.3] × 10^−7^ (2.8)
*msh3*Δ *msh6*Δ*^a^*	4234	3.1 [1.9–4.8] × 10^−6^ (2,040)	4.8 [3.9–10.6] × 10^−5^ (5,630)	3.1 [1.7–4.6] × 10^−6^ (80.1)
*msh3*Δ *msh6-K988A*	9385	3.65 [0.37–4.73] × 10^−6^ (2,400)	6.99 [1.28–10.3] × 10^−5^ (8,190)	2.56 [1.00–3.69] × 10^−6^ (66.1)
*msh3*Δ *msh6-K988M*	9387	5.04 [4.40–37.2] × 10^−6^ (3,320)	9.48 [6.55–50.3] × 10^−5^ (11,100)	3.60 [2.42–22.9] × 10^−6^ (93.0)
*msh3*Δ *msh6-K988R*	9386	4.79 [2.84–7.74] × 10^−6^ (3,150)	9.23 [6.62–11.8] × 10^−5^ (10,800)	2.48 [2.00–3.96] × 10^−6^ (64.1)

*^a^* Rates from Shell *et al.* (78).

### The msh2 and msh6 Walker A motif mutations are not dominant

A genetic screen for dominant mutations in *MSH6* recovered several *msh6* mutations that affected residues clustered around the Msh6 ATPase site (51). These mutations cause large increases in mutation rates, including increased frameshift reversion rates when present as single mutations at the *MSH6* locus and when present on a low-copy *ARS CEN* plasmid in an otherwise WT strain (21, 30, 51, 52). Therefore, we tested whether the Walker A Lys mutations were dominant alleles by transforming low-copy number *ARS CEN* plasmids containing the different *MSH2* or *MSH6* alleles into a WT strain. In contrast to the previously identified dominant mutations like *msh6-G1142D* and *msh6-S1036P* (21, 30, 51, 52), none of the plasmid-borne *MSH2* or *MSH6* Walker A Lys mutations caused an increase in mutation rates in a WT strain and were therefore not dominant to the chromosomally encoded *MSH2* or *MSH6* genes, respectively (Fig. S1). Previous analysis of the Walker A Gly *msh2* mutations showed that these mutations did not cause an increased mutation rate in a WT strain when present on an *ARS CEN* plasmid (46, 47), which is consistent with our results, but did cause a very small increase in mutation rate when present in a heterozygous diploid *S. cerevisiae* strain. These mutations and a Walker A Gly *msh6* mutation were dominant when overexpressed using a *GAL10* promoter on a high-copy-number 2-μm plasmid (28, 46, 47). Our results and those of others (28, 46, 47, 49) are most consistent with Walker A mutations resulting in nonfunctional Msh2 or Msh6 proteins. Therefore, overexpression of the nonfunctional Msh2 Walker A Gly mutant protein may inactivate MMR by sequestering all of the WT Msh6 and Msh3 proteins in inactive Msh2–Msh6 and Msh2–Msh3 complexes, and overexpression of the nonfunctional Msh6 Walker A Gly mutant protein may inactivate MMR by sequestering all of the WT Msh2 protein in inactive Msh2–Msh6 complexes rather than blocking MMR due to some type of gain-of-function defect similar to those caused by dominant mutations like *msh6-G1142D* and *msh6-S1036P* (21, 30, 51, 52).

### Msh2 and Msh6 Walker A motif Lys mutant complexes are partially defective in reconstituted MMR reactions in vitro

**Figure 2.**
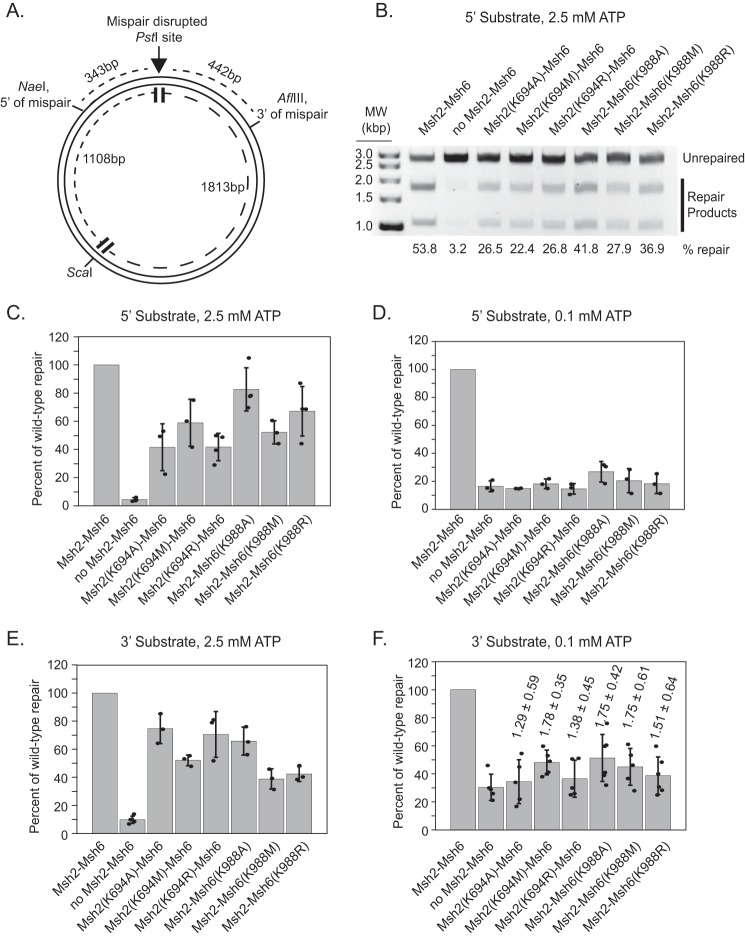
**Msh2 and Msh6 mutants are partially defective in an *in vitro* reconstituted MMR assay.** *A*, schematic representation of the DNA substrate for the reconstituted repair assay. Substrates had a nick at either the NaeI or AflIII site but not both. *B*, representative gel showing repair of the 5′ nicked NaeI substrate used in an assay with 2.5 mm ATP. *C–F*, the amount of repair relative to WT Msh2–Msh6 was quantitated for the 5′ nicked NaeI substrate at 2.5 mm ATP (100% repair = 43.5% of substrate repaired) (*C*), the 5′ nicked NaeI substrate at 0.1 mm ATP (100% repair = 17.8% of substrate repaired) (*D*), the 3′ nicked AflIII substrate at 2.5 mm ATP (100% repair = 23.2% of substrate repaired) (*E*), or the 3′ nicked AflIII substrate at 0.1 mm ATP (100% repair = 12.1% of substrate repaired) (*F*) by normalizing the percentage of repair in each experiment to the WT levels and averaging a minimum of at least three independent experiments. *Error bars* represent the S.D. from the mean, and individual values from different experiments are indicated by the *dots* on each histogram *bar*. In *F*, *numbers* over the histogram *bars* are the average of the ratio of mutant repair to repair without Msh2–Msh6 (compared internally between experiments) with the *error* indicating the S.D.

We next tested whether the Walker A Lys mutant Msh2–Msh6 complexes could function in MMR reactions reconstituted *in vitro* with purified proteins and DNA substrates containing a single thymine base insertion mispair on the same strand as a pre-existing nick that is either 5′ of the mispair at the NaeI site (5′ repair assay) or 3′ of the mispair at the AflIII site (3′ repair assay) (Fig. 2*A*). Nicked strand–specific mispair correction is Msh2–Msh6-dependent and restores a PstI site, which is detected by restriction endonuclease digestion with both PstI and ScaI, resulting in two repair-specific DNA species (Fig. 2*B*) (58). The 5′ nick–directed repair reactions contain ATP, Mg^2+^, WT or mutant Msh2–Msh6, replication protein A, Exo1, DNA polymerase δ, PCNA, RFC-Δ1N (RFC-Δ1N is an RFC1–5 complex containing an N-terminal truncation of RFC1 that reduces its DNA binding but leaves all other functions intact (59); the 3′ nick–directed repair assay reactions in addition contained Mlh1–Pms1. Previous studies have demonstrated that the 5′ nick–directed repair assay depends on Exo1 but does not require Mlh1–Pms1 (53, 58, 60), whereas the 3′ nick–directed repair assay depends on Mlh1–Pms1 (53, 60) and under some conditions can be at least partially independent of Exo1 (61).

We first investigated the ability of the Walker A Lys mutant Msh2–Msh6 complexes to support repair in the 5′ nick–directed repair assay. In these reactions, Exo1 initiates excision at the 5′ nick in a reaction that is significantly stimulated by Msh2–Msh6 in a mispair recognition–dependent fashion resulting in excision of the mispair (58, 62). The resulting single-stranded DNA gap is then filled in by DNA polymerase δ and accessory proteins; in addition, DNA polymerase ϵ can substitute for DNA polymerase δ (60). At concentrations of ATP (2.5 mm) that exceed the *K_d_* for ATP binding by both the Msh2 and Msh6 nucleotide-binding sites, the Walker A Lys mutant Msh2–Msh6 complexes supported 40–75% of the level of repair supported by WT Msh2–Msh6 (Fig. 2, *B* and *C*). In contrast, all of these mutant complexes were completely defective at low ATP concentrations (0.1 mm), which was still sufficient for MMR supported by WT Msh2–Msh6 (Fig. 2*D*), albeit at a somewhat reduced efficiency (17.8% of total DNA repaired at 0.1 mm ATP compared with 43.5% at 2.5 mm ATP).

We next investigated the ability of the Walker A Lys mutant Msh2–Msh6 complexes to support repair in the 3′ nick–directed repair assay (53, 60, 63, 64). This reaction involves 1) mispair recognition by Msh2–Msh6; 2) recruitment of Mlh1–Pms1, which produces strand-specific nicks 5′ of the mispair on the prenicked DNA strand; 3) excision of the mispair by Exo1 from the Mlh1–Pms1-generated nicks; and 4) gap-filling resynthesis by DNA polymerase δ and accessory proteins. With WT Msh2–Msh6, these reactions resulted in repair of ∼26% of the 3′ nicked DNA substrate at high ATP concentrations (2.5 mm) (Fig. 2*E*). In contrast, the substitution of the six Walker A Lys mutant Msh2–Msh6 complexes for WT Msh2–Msh6 resulted in between 40 and 75% of WT levels of repair at 2.5 mm ATP (Fig. 2*E*). Reducing the ATP concentration to 0.1 mm decreased the overall level of repair by WT Msh2–Msh6 to ∼13%. Interestingly, reactions containing Msh2(K694A)–Msh6, Msh2(K694R)–Msh6, and Msh2–Msh6(K988M) mutants showed reduced levels of repair at 0.1 mm ATP that were possibly not above the background levels of repair observed in the absence of Msh2–Msh6 (Fig. 2*F*). In contrast, reactions containing Msh2(K694M)–Msh6, Msh2–Msh6(K988A), and Msh2–Msh6(K988M) all resulted in low levels of repair at 0.1 mm ATP that were above background levels of repair (Fig. 2*F*).

Because low ATP conditions (0.1 mm) eliminated 5′ nick–directed repair supported by all the Walker A Lys mutant Msh2–Msh6 complexes (Fig. 2*D*) but did not completely eliminate Mlh1–Pms1–dependent 3′ nick–directed repair supported by all the Walker A Lys mutant Msh2–Msh6 complexes (Fig. 2*F*), we hypothesized that MMR under low ATP conditions in reactions containing Mlh1–Pms1 might be independent of Exo1. We therefore tested the 3′ nick–directed repair assay with and without Exo1. Consistent with the hypothesis that repair in the 3′ nick–directed repair assay under low ATP conditions was Exo1-independent, the ratios of the percentage of repair without Exo1 to the percentage of repair with Exo1 were 0.92 ± 0.16, 0.97 ± 0.08, and 1.23 ± 0.13 for the WT Msh2–Msh6 and Msh2(K694M)–Msh6 and Msh2–Msh6(K988M) mutant complexes, respectively. That these ratios are close to 1 suggests that absence of Exo1 does not reduce the level of repair observed under these conditions. Together, these results show that the six Walker A Lys mutant Msh2–Msh6 complexes are partially defective but largely functional for both the 5′ and 3′ nick–directed repair at physiological ATP concentrations (2.5 mm ATP). However, they appear to be completely defective for Exo1-dependent repair at low ATP concentrations. Interestingly, the largely functional repair seen at physiological ATP concentrations (2.5 mm ATP) contrasts with the complete MMR defect exhibited by these mutant Msh2–Msh6 complexes *in vivo* (Table 1).

### The Walker A Lys mutant Msh2–Msh6 complexes are proficient for mispair binding

**Figure 3.**
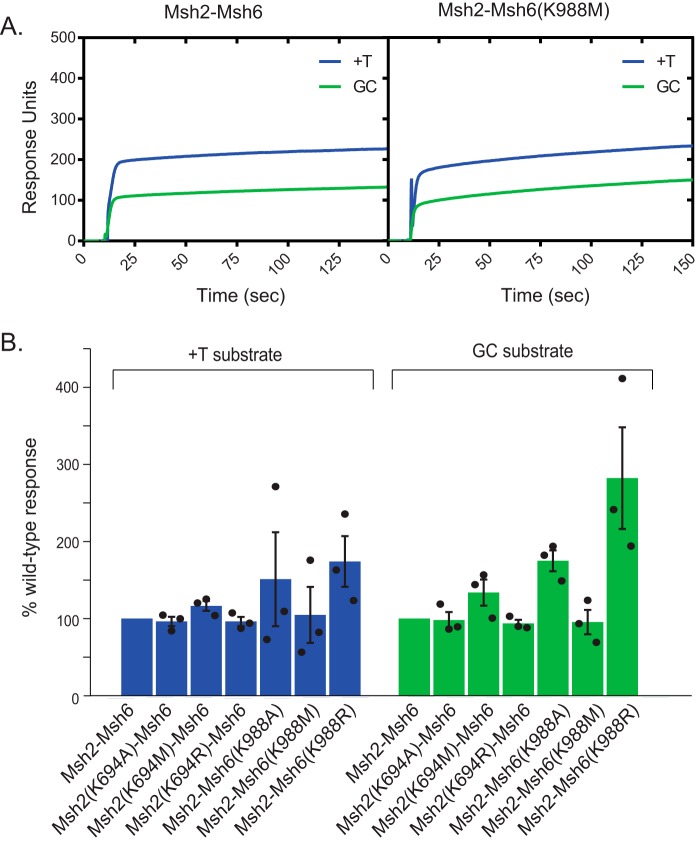
**Analysis of Msh2–Msh6-mispair binding in the absence of nucleotide.** *A*, representative SPR sensorgrams showing WT Msh2–Msh6 or Msh2–Msh6(K988M) binding to mispaired (+T; *blue*) or base-paired (GC; *green*) DNA in the absence of ATP. *B*, plot of the percentage of WT Msh2–Msh6 steady-state response (RU_max_) for the +T mispair–containing substrate (*left*, *blue*) and the GC fully base-paired control substrate (*right*, *green*); the level of binding was normalized to the WT level of binding, which was set at 100%. In all cases, the *R*^2^ values for the fits to determine RU_max_ were above 0.993, and in most cases, the *R*^2^ values for the fits were above 0.999. *Error bars* represent the S.D. from the mean, and individual values from different experiments are indicated by the *dots* on each histogram *bar*.

The ability of the Walker A Lys mutant Msh2–Msh6 complexes to support reconstituted MMR reactions suggests that these complexes can recognize mispair-containing DNA. To directly assess mispair binding, we used surface plasmon resonance (SPR) to measure the binding of the mutant complexes to 236-bp dsDNA molecules with and without a central +T insertion mispair in the absence of ATP. DNA molecules were immobilized at one end on a streptavidin-coated surface through a 5′-biotin located at the end of one of the two DNA strands. Representative sensorgrams monitoring response units (RU) of binding revealed that both WT Msh2–Msh6 and Msh2–Msh6(K988M) complexes preferentially bound the +T-containing substrate relative to the control DNA lacking a mispair and that both complexes had similar levels of binding (Fig. 3*A*). We determined the steady-state responses by fitting the association curves (RU_max_; see “Experimental procedures”). All of the Msh2 and Msh6 Walker A Lys mutant Msh2–Msh6 complexes exhibited levels of mispair binding that were the same as that of WT Msh2–Msh6 (Fig. 3*B*), indicating that all of the mutant complexes were proficient for mispair binding in the absence of added nucleotides. Moreover, the Msh2(K694M)–Msh6, Msh2–Msh6(K988A), and Msh2–Msh6(K988R) complexes had modestly increased levels of binding to both mispaired and fully base-paired DNAs relative to WT Msh2–Msh6 (Fig. 3*B*). These results are consistent with the results of previous experiments in which mispair binding by a limited subset of Walker A and B mutant Msh2–Msh6 complexes was assessed using gel shift and filter binding assays (28, 46–49).

### Msh2 and Msh6 Walker A Lys mutant complexes are defective for sliding clamp formation

**Figure 4.**
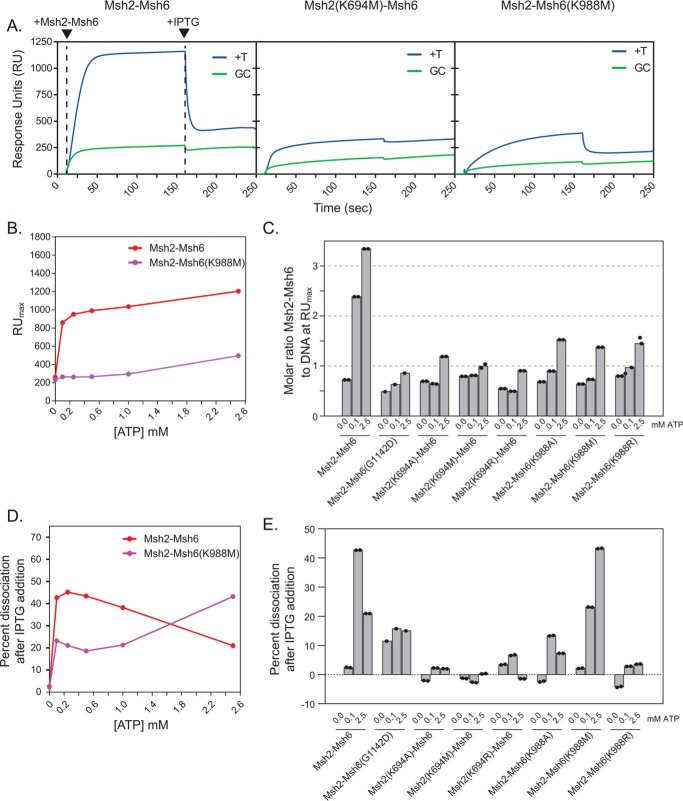
**Msh2 and Msh6 mutants have mispair binding defects in the presence of ATP and are affected by ATP differentially from WT Msh2–Msh6.** *A*, representative sensorgram showing substantial or little sliding clamp formation by WT Msh2–Msh6, Msh2(K694M)–Msh6, and Msh2–Msh6(K988M) on +T mispair–containing substrate (*blue*) but little sliding clamp formation on fully base-paired substrate (*green*). Msh2–Msh6 was added at 10 s, and IPTG was added at 160 s. *B*, fitted steady-state response (RU_max_) of WT Msh2–Msh6 (*red*) and Msh2–Msh6(K988M) (*purple*) on a +T mispair–containing substrate is plotted as a function of ATP concentration. In all cases, fits to the association curves had *R*^2^ values greater than 0.996. *C*, the molar ratio of Msh2–Msh6 complexes to DNA at steady state (RU_max_) was calculated for WT and mutant Msh2–Msh6 complexes at different ATP concentrations. In all cases, fits to the association curves had *R*^2^ values greater than 0.996. *D*, the percentage of Msh2–Msh6 dissociation for WT Msh2–Msh6 (*red*) and Msh2–Msh6(K988M) (*purple*) at 200 s (corresponding to 40 s or >10 half-lives for the WT sliding clamp after the addition of IPTG) is plotted as a function of ATP concentration. *E*, the percentage of Msh2–Msh6 dissociation was determined for WT and mutant Msh2–Msh6 complexes at different ATP concentrations. In *C* and *E*, individual values from different experiments are indicated by the *dots* on each histogram *bar*.

Mispair-bound Msh2–Msh6 is converted into a sliding clamp after binding ATP in both the Msh2 and Msh6 nucleotide-binding sites (22–24, 32), and mutations in *MSH2* and *MSH6* that result in Msh2–Msh6 complexes that cannot form sliding clamps result in MMR defects (21, 30, 51). Formation of ATP-dependent Msh2–Msh6 sliding clamps can be observed in SPR experiments by comparing the level of binding of Msh2–Msh6 to mispair-containing DNAs that are blocked at either one or both ends. Double end–blocked substrates allow a dramatic increase in RU due to a buildup of Msh2–Msh6 complexes because sliding clamps slide away from the mispair but remain trapped by the end blocks, allowing additional Msh2–Msh6 complexes to bind to the mispair and form sliding clamps. In contrast, Msh2–Msh6 sliding clamps slide off the end of single end–blocked DNAs, and hence the single end–blocked substrates do not accumulate large amounts of bound Msh2–Msh6 complexes. Furthermore, if Msh2–Msh6 sliding clamps accumulate on double end–blocked mispaired DNA and one of the end blocks is released, the sliding clamps rapidly dissociate from the DNA by sliding off the free DNA end, providing additional evidence for the formation of sliding clamps. We have previously developed a system involving one stable end block that immobilizes the 236-bp mispair-containing DNA to the surface (biotin-streptavidin) and a proximal reversible end block (*lacO*-LacI) (22). Thus, in the presence of ATP, a dramatic increase in RU due to sliding clamp formation can be observed on the double end–blocked substrate, whereas a rapid decrease in RU is observed upon IPTG addition, which releases the LacI end block and allows the majority of accumulated Msh2–Msh6 complexes to slide off the substrate. Examples of this type of SPR analysis for the WT Msh2–Msh6, the sliding clamp–defective Msh2(K694M)–Msh6, and the partially sliding clamp–defective Msh2–Msh6(K688M) complexes are shown in Fig. 4*A*.

We first analyzed the steady-state binding of Msh2–Msh6 on double end–blocked mispaired DNA as a function of ATP concentration (Fig. 4*B*). WT Msh2–Msh6 showed a high level of steady-state binding (RU_max_) at the lowest ATP concentration tested (0.1 mm), which was >3-fold higher than the level of binding in the absence of ATP, and a gradual increase in steady-state binding up to the highest ATP concentration of 2.5 mm (Fig. 4*B*). Because responses in SPR are proportional to bound mass (65), we calculated the steady-state molar ratio of Msh2–Msh6 at RU_max_ based on the initial RU response due to DNA. For WT Msh2–Msh6, 2.4–3.3 complexes of WT Msh2–Msh6 complexes were bound per DNA molecule (Fig. 4*C*). In contrast, the Msh2–Msh6(G1142D) dominant mutant complex, included as a control, was largely unaffected by ATP concentration and showed no more than 0.86 complex bound per mispaired DNA molecule at 0.1 and 2.5 mm ATP, respectively (Fig. 4*D*), consistent with previous results indicating that this complex cannot efficiently form sliding clamps (30). Like Msh2–Msh6(G1142D), steady-state binding of the Walker A Lys mutant Msh2–Msh6 complexes was largely insensitive to ATP, although binding was highest at the highest ATP concentration tested (2.5 mm). The Msh2-Lys mutant Msh2–Msh6 complexes had a maximum binding of ∼1 Msh2–Msh6 complexes per mispaired DNA molecule, whereas the Msh6-Lys mutant Msh2–Msh6 complexes had a maximum binding of up to 1.4 Msh2-Msh6 complexes bound per mispaired DNA (Fig. 4, *A*, *B*, and *C*).

We then analyzed the dissociation of Msh2–Msh6 after addition of 1 mm IPTG to release the LacI end block. The half-life of the major component of dissociation for WT Msh2–Msh6 in the presence of ATP was found to be 3.9 ± 0.1 s (see “Experimental procedures”). We therefore measured the percentage of dissociation of this rapid component by comparing the response units before and after adding IPTG. The postaddition time point was 200 s, which corresponded to ∼10 half-lives. In the absence of ATP, WT Msh2–Msh6 was insensitive to the addition of IPTG (2.4% dissociation), but showed dramatically increased dissociation at different ATP concentrations (Fig. 4*D*). In contrast, Msh2–Msh6(K988M), which showed some ATP-dependent increased accumulation of double end–blocked mispaired DNA and some IPTG-induced rapid dissociation in the presence of ATP (Fig. 4*A*), had reduced levels of dissociation at all ATP concentrations below the maximum concentration tested (2.5 mm ATP; Fig. 4*D*). As a control, we examined the Msh2–Msh6(G1142D) dominant mutant complex that is incapable of sliding clamp formation (21, 30, 51, 52) and found that the dissociation upon addition of IPTG was unaffected by addition of ATP. Most of the Walker A Lys mutant Msh2–Msh6 complexes showed little or no evidence of IPTG-induced dissociation in the presence of ATP. In contrast, the low (compared with WT Msh2–Msh6; Fig. 4*A*) amount of Msh2–Msh6(K988M) complex that accumulated on double end–blocked mispaired DNA in the presence of ATP showed IPTG-induced dissociation (Fig. 4, *A*, *D*, and *E*). Overall, the results of the two types of experiments are consistent with the view that the Walker A Lys mutant Msh2–Msh6 complexes bind mispairs and either do not form ATP-induced sliding clamps that ultimately accumulate on the mispaired DNA or, in the case of the Msh2–Msh6(K988M) mutant complex, form a significantly reduced level of sliding clamps. These results are consistent with previous observations that the limited number of *S. cerevisiae* Walker A and B motif Msh2–Msh6 mutant complexes analyzed showed normal mispair binding in the absence of nucleotides in gel shift and filter binding assays and bound to mispairs in these assays in the presence of ATP (28, 46, 47, 49, 50). We note that the human Walker A Lys to Ala mutant MSH2–MSH6 complexes showed that the mutant MSH2–MSH6 complexes also displayed sliding clamp defects although not as striking as the defects seen here (37).

### Walker A Lys mutant Msh2–Msh6 complexes show reduced direct dissociation

**Figure 5.**
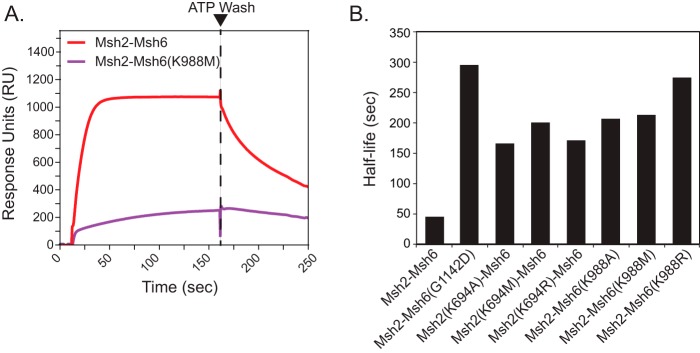
**Analysis of Msh2–Msh6 directly dissociating from double end–blocked DNA.** *A*, representative surface plasmon resonance sensorgrams showing WT and mutant Msh2–Msh6 binding to, and directly dissociating from, mispaired DNA in the presence of ATP. Direct dissociation reactions (*red* and *purple* curves) were performed the same as the sliding clamp formation experiments except IPTG and Msh2–Msh6 were omitted from the ATP wash step. Consequently, any Msh2–Msh6 leaving the DNA must do so directly due to the continual presence of the LacI end block. *B*, half-lives of the major component of dissociation of WT and mutant Msh2–Msh6 complexes from double end–blocked DNA. In all cases, fits to the dissociation curves had *R*^2^ values greater than 0.997.

Msh2–Msh6 complexes can directly dissociate from mispaired DNA in addition to sliding off the ends of the DNA. We tested direct dissociation of mispair-bound Msh2–Msh6 using the 236-bp biotin-streptavidin and *lacO*-LacI double end–blocked substrate, which eliminates the faster end-dependent dissociation of sliding clamps. Msh2–Msh6 was first bound to the DNA in buffer containing ATP and LacI. Buffer flow was then switched to buffer containing ATP and LacI but lacking Msh2–Msh6. WT Msh2–Msh6 showed a faster dissociation from the DNA than the Walker A Lys mutant Msh2–Msh6 complexes and the Msh2–Msh6(G1142D) complex (Fig. 5). The dissociation curves were fit (see “Experimental procedures”), and the major component of the dissociation of WT Msh2–Msh6 had a half-life of 45.3 s, which was 11.6-fold slower than the end-dependent dissociation of WT Msh2–Msh6 (3.9 s). In contrast, the half-lives the major component of dissociation of the Msh2–Msh6(G1142D) complex and the Walker A Lys mutant Msh2–Msh6 complexes were substantially longer, ranging from 165 to 294 s (Fig. 5*B*). We also examined the direct dissociation of WT Msh2–Msh6 in the absence of nucleotide, conditions where Msh2–Msh6 binds mispairs but does not form sliding clamps; under these conditions, the major component of dissociation of the WT Msh2–Msh6 complex had a half-time of dissociation of 740 s. These results are consistent with the idea that the mispair-bound complexes are more stably associated with DNA than Msh2–Msh6 clamps that are sliding on DNA and are not bound to a mispair.

### Walker A Lys mutant Msh2–Msh6 complexes are proficient for recruiting Mlh1–Pms1

**Figure 6.**
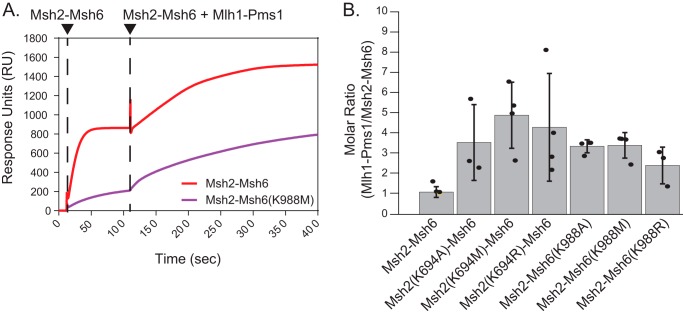
**Msh2 and Msh6 mutants can recruit Mlh1–Pms1 to mispaired DNA.** *A*, representative SPR sensorgram for Mlh1–Pms1 recruitment on GT mispair–containing DNA by WT Msh2–Msh6 (*red*) or Msh2–Msh6(K988M) (*purple*); Mlh1–Pms1 addition is indicated by the *rightmost vertical dotted line. B*, molar ratios were calculated by determining the increase in RU_max_ due to Msh2–Msh6 and Mlh1–Pms1 from fits to the association curves (see “Experimental procedures”) and by scaling these responses by the molecular weights of the Msh2–Msh6 and the Mlh1–Pms1 complexes. In all cases, fits to the association curves had *R*^2^ values greater than 0.998. *Error bars* represent the S.D. from the mean, and individual values from different experiments are indicated by the *dots* on each histogram *bar*.

In addition to being required for the formation of Msh2–Msh6 sliding clamps, ATP is also required for Msh2–Msh6 to recruit Mlh1–Pms1 (22, 66–68). To monitor Mlh1–Pms1 recruitment using SPR, Msh2–Msh6 and LacI in buffer containing ATP was flowed over the double end–blocked mispaired DNA-bound surface until binding reached steady state, and then the buffer flow was switched to buffer containing Msh2–Msh6, Mlh1–Pms1, LacI, and ATP; Mlh1–Pms1 recruitment was observed as an increase in RU relative to the steady-state level of Msh2–Msh6 binding alone (Fig. 6*A*). The increase in RU after the addition of Mlh1–Pms1 is thought to be due entirely to Mlh1–Pms1 and not additional Msh2–Msh6 (22), which is consistent with single-molecule studies of the bacterial MutS and MutL homologs (36). WT Msh2–Msh6 recruited Mlh1–Pms1 to a similar extent as observed previously (21, 22, 30, 31), and all six Walker A Lys mutant Msh2–Msh6 complexes were also able to recruit Mlh1–Pms1 (Fig. 6, *A* and *B*). The molecular ratio for the recruitment of Mlh1–Pms1 to WT Msh2–Msh6 was 1.07 (Fig. 6*B*), which is consistent with the formation of a 1:1 complex of Mlh1–Pms1 to Msh2–Msh6. Each of the Walker A Lys mutant Msh2–Msh6 complexes clearly bound Mlh1–Pms1 with the observed molecular ratios ranging from 2.06 to 3.75 Mlh1–Pms1 complexes bound per Msh2–Msh6 complex (Fig. 6*B*). These results indicate that the mispair-bound Walker A Lys mutant Msh2–Msh6 complexes are proficient for recruiting Mlh1–Pms1. However, the altered (increased) ratio of Mlh1–Pms1 to Msh2–Msh6 seen for the mutant Msh2–Msh6 complexes compared with WT Msh2–Msh6 suggests that some aspect of the dynamics of Mlh1–Pms1 recruitment is altered by the Walker A Lys amino acid substitutions. The ability of the Walker A Lys mutant Msh2–Msh6 complexes to recruit Mlh1–Pms1 is distinct from the properties of the previously characterized dominant *MSH6* mutant Msh2–Msh6 complexes: both types of mutant complexes bind mispairs but do not form sliding clamps, whereas the dominant mutant Msh2–Msh6 complexes either fail to recruit Mlh1–Pms1 or in the case of Msh2–Msh6(G1142D) recruit much reduced amounts of Mlh1–Pms1 (30).

### Msh2 and Msh6 Walker A Lys mutant complexes are partially defective for activation of the Mlh1–Pms1 endonuclease

**Figure 7.**
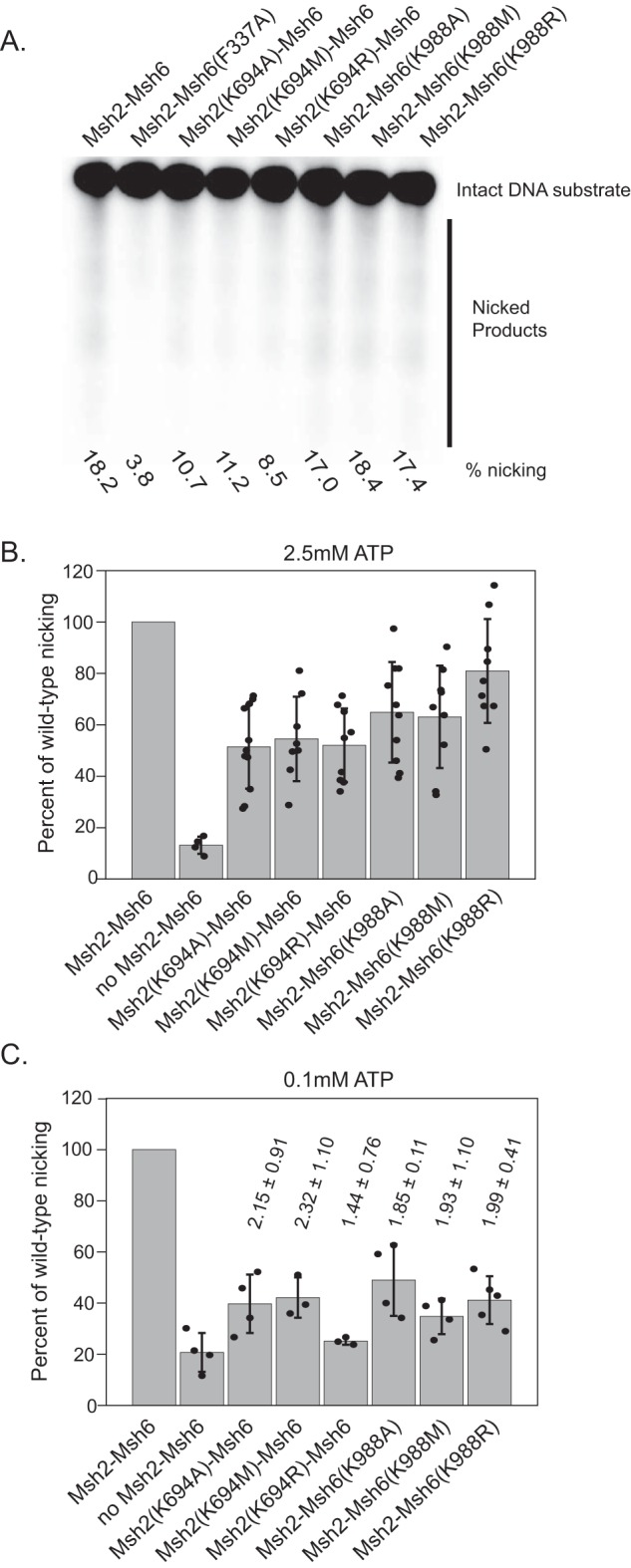
**Msh2 and Msh6 mutants are partially defective for Mlh1–Pms1 endonuclease activation.** Assays contain WT or mutant Msh2–Msh6, Mlh1–Pms1, PCNA, RFC-Δ1N, a circular DNA substrate with a mispair and nick 3′ to the mispair at the AflIII site (see Fig. 2*A* for schematic of DNA substrate), and 0.1 or 2.5 mm ATP. *A*, representative Southern blot showing levels of nicking promoted by WT or mutant Msh2–Msh6 at 2.5 mm ATP. *B* and *C*, quantitation of reactions containing 2.5 mm ATP (100% nicking = 22.6% of substrate nicked) (*B*) and 0.1 mm ATP (100% nicking = 17.2% of substrate nicked) (*C*) were performed by normalizing the percentage of repair in each experiment to the WT levels and averaging a minimum of at least three independent experiments. *Error bars* represent the S.D. from the mean, and individual values from different experiments are indicated by the *dots* on each histogram *bar*. In *C*, *numbers* over the histogram *bars* are the average of the ratio of mutant nicking to nicking without Msh2–Msh6 (compared internally between experiments) with the *error* indicating the S.D.

The Mlh1–Pms1 endonuclease activity that is activated in an Msh2–Msh6-, PCNA-, RFC-, and ATP-dependent manner is essential for MMR *in vivo* and for 3′ nick–directed MMR in reconstituted MMR reactions *in vitro* (53, 60, 63, 64). We examined the Walker A Lys mutant Msh2–Msh6 complexes for their ability to promote Mlh1–Pms1 endonuclease activity in an assay containing a mispaired DNA substrate with a pre-existing 3′ nick at the AflIII site (Fig. 2*A*). The reaction products were analyzed by linearizing the DNA with ScaI followed by alkaline agarose gel electrophoresis and Southern blotting using a probe that hybridizes just 3′ of the diagnostic ScaI site on the 3′ nick–containing strand and reveals the position of the Mlh1–Pms1-induced nick closest to the ScaI site (53). Consistent with previous results (53), in the presence of WT Msh2–Msh6 in reactions containing 2.5 mm ATP, ∼22% of the input substrate was nicked between the ScaI and AflIII sites as revealed by a smear of DNA fragments that were shorter than the 1.55-kb starting fragment (Fig. 7*A*). In contrast, omitting Msh2–Msh6 or substituting the mispair binding–defective Msh2–Msh6(F337A) mutant resulted in substantially less nicking by Mlh1–Pms1 (Fig. 7, *A* and *B*). The Walker A Lys mutant Msh2–Msh6 complexes were largely functional for promoting Mlh1–Pms1 nicking with the Msh2 mutants promoting ∼50% of the nicking of WT Msh2–Msh6 and the Msh6 mutants promoting ∼60–80% of WT activity at 2.5 mm ATP (Fig. 7, *A* and *B*). When the ATP in the nicking assay was reduced to 0.1 mm, we observed that all the mutants showed further reduced nicking that was in all cases, except the Msh2(K694R)–Msh6 mutant, higher than the background level of nicking seen in the absence of Msh2–Msh6 (Fig. 7*C*). These results indicate that the Walker A Lys mutant Msh2–Msh6 complexes are at worst only partially defective for promoting activation of the Mlh1–Pms1 endonuclease relative to that seen with WT Msh2–Msh6, especially at physiological ATP concentrations (2.5 mm ATP (69, 70)).

## Discussion

ATP binding and hydrolysis by the Msh2–Msh6 complex are linked to multiple steps in MMR (22–24, 41). Here, we mutated the conserved Walker A motif lysine codons (Lys^694^ in Msh2 and Lys^988^ in Msh6) of *MSH2* and *MSH6* to alanine, arginine, or methionine codons and studied the mutations and resulting mutant Msh2–Msh6 complexes in detail. All six mutations caused ATP binding defects at one or both nucleotide-binding sites and caused complete MMR defects *in vivo*. In contrast, the same mutant proteins were partially or largely functional in reconstituted MMR reactions *in vitro* as well as in biochemical assays of individual MMR steps. Of all the biochemical defects observed *in vitro*, the inability of the Walker A Lys mutant Msh2–Msh6 complexes to form sliding clamps displayed the best correlation with the complete MMR defects caused by the Walker A Lys mutations *in vivo*.

Previous studies of different families of Walker A ATPases have shown that changing the critical invariant Walker A motif Lys residue to alanine or methionine often results in ATP binding defects, whereas changing the Lys residue to arginine can cause ATP hydrolysis defects (24, 56). Contrary to initial expectations, UV cross-linking experiments showed that all six Walker A Lys amino acid substitutions tested caused an ATP binding defect at the mutated nucleotide-binding site (Fig. 1). As a result, we did not examine the ATP hydrolysis properties of the mutant Msh2–Msh6 complexes; indeed, previous studies of the Msh2(K694A)–Msh6 and Msh2(K694R)–Msh6 complexes have shown that ATP binding defects also result in altered ATP hydrolysis (27). These results are similar to those observed with the human MSH2 Walker A lysine to arginine substitution that caused a strong ATP binding defect seen by UV cross-linking (49). The human MSH6 lysine to arginine substitution caused a small ATP binding defect in UV cross-linking experiments in contrast to our results (49). Interestingly, two of the Msh2 mutant complexes (Msh2(K694A)–Msh6 and Msh2(K694R)–Msh6) had ATP binding defects at both nucleotide-binding sites, which is consistent with previous observations that nucleotide binding at one of the Msh2–Msh6 nucleotide-binding sites can affect nucleotide binding and hydrolysis at the other nucleotide-binding site (21, 24, 27, 55). Overall, these results suggest that the defects associated with the Walker A Lys mutant Msh2–Msh6 complexes primarily reflect ATP binding defects and altered conformational change–driven protein dynamics due to reduced ATP binding.

All of the Walker A Lys mutant Msh2–Msh6 complexes were proficient for mispair binding and proficient for recruitment of Mlh1–Pms1. In contrast, all six of the Walker A Lys mutant Msh2–Msh6 complexes displayed similar biochemical defects in three different MMR-related assays: 1) repair in 5′ nick–directed Exo1-dependent MMR (Fig. 2, *B*, *C*, and *D*), 2) repair in 3′ nick–directed Mlh1–Pms1–dependent MMR (Fig. 2, *E* and *F*), and 3) activation of the Mlh1–Pms1 endonuclease (Fig. 7). The extent of the defect depended on ATP concentration with small partial defects being observed at physiological 2.5 mm ATP (69, 70) and stronger defects being seen at less than physiological 0.1 mm ATP. At 0.1 mm ATP, all of the Walker A Lys mutant Msh2–Msh6 complexes showed a complete defect in 5′ nick–directed Exo1-dependent MMR but only a partial defect in 3′ nick–directed Exo1-independent MMR. This result suggests that the Walker A Lys mutant Msh2–Msh6 complexes are unable to support excision by Exo1 at low ATP concentrations, potentially due to defects in the recruitment of Exo1 (71–73). Consistent with this, we have identified a mutation altering an amino acid in the Msh2 nucleotide-binding region that causes a defect in Exo1-dependent MMR and causes a partial defect in recruitment of Exo1 by Msh2 (71, 74). We have also shown that tethering Exo1 to the Msh2–Msh6 complex to incorporate it into the Msh2–Msh6 sliding clamp can overcome defects in Exo1 recruitment by Msh2 (71). Together, these results suggest that Exo1 recruitment by Msh2–Msh6 may be dependent upon ATP-induced conformational changes that occur during sliding clamp formation.

We previously identified dominant mutations in *MSH6* that cause a large increase in mutation rates in frameshift reversion assays in contrast to *msh6*Δ mutations that only cause small increases in mutation rates in frameshift reversion assays due to the redundancy between *MSH6* and *MSH3*. The dominant *MSH6* mutant Msh2–Msh6 complexes bind mispairs but do not form sliding clamps and either fail to recruit Mlh1–Pms1 or recruit reduced amounts of Mlh1–Pms1, suggesting that the dominant mutant Msh2–Msh6 complexes bind mispairs and then occlude Msh2–Msh3 from binding mispairs. This raises the question of how is it that the *msh6* Walker A Lys mutations are not dominant given that they result in mutant Msh2–Msh6 complexes that bind mispairs but do not form sliding clamps. One possibility is that when mispair-bound Walker A Lys mutant Msh2–Msh6 complexes recruit Mlh1–Pms1, the mutant Msh2–Msh6 complexes are induced to form sliding clamps. This would result in increased recruitment of both Msh2–Msh6 and Mlh1–Pms1, consistent with our data, and would then prevent the Walker A mutant Msh2–Msh6 complexes from occluding mispairs *in vivo*. This would also account for the ability of the mutant complexes to largely support MMR in reconstituted MMR reactions and activation of the Mlh1–Pms1 endonuclease *in vitro*, further implying that sliding clamp formation is required for these activities. Testing this hypothesis will likely require single-molecule analysis that is beyond the scope of the present study (41, 73).

The ability of the mispair-bound Walker A Lys mutant complexes to recruit Mlh1–Pms1 and possibly other proteins like Exo1 appears to account for their ability to support MMR and Mlh1–Pms1 endonuclease activation to a large extent *in vitro*, especially at physiological ATP levels (69, 70). However, this raises the question of which defect underlies the complete MMR defect caused by the Walker A Lys mutations *in vivo*. All of the Walker A Lys mutant Msh2–Msh6 complexes had defects in sliding clamp formation, and only the Msh2–Msh6(K988M) complex displayed any end-dependent dissociation of the bound complex, similar to WT Msh2–Msh6. The mispair-bound Walker A Lys mutant Msh2–Msh6 complexes also showed reduced turnover from double end–blocked mispaired DNA compared with WT Msh2–Msh6, which is consistent with mispair-bound Msh2–Msh6 being more stably bound to the mismatch than Msh2–Msh6 sliding clamps on base-paired DNA. One hypothesis that could explain the contrasting observations *in vivo* and *in vitro* is that a signal amplification mechanism in which the formation of multiple sliding clamps on mispaired DNA, as proposed previously, is required to recruit multiple molecules of downstream MMR factors (43, 75). This would ensure efficient MMR *in vivo*, which may involve displacement of nucleosomes and prevention of the processing of replication intermediates (45, 71, 76, 77). In this model, the inability of the Walker A mutant Msh2–Msh6 complexes to form multiple sliding clamps leads to an MMR defect *in vivo*.

## Experimental procedures

### Strains and plasmids

*S. cerevisiae* strains were grown at 30 °C in either standard YPD medium (1% yeast extract (Fisher Chemical), 2% peptone (Fisher Chemical), and 2% dextrose (Fisher Chemical)) or the appropriate synthetic dropout medium (0.67% yeast nitrogen base (Difco), 2% dextrose, and 0.2% amino acid dropout mixture (US Biological)) where appropriate. All *S. cerevisiae* strains were derived from the S288C strain background. *Escherichia coli* overexpression strains were grown at 37 °C in LB (10 g of tryptone (Fisher Chemical), 5 g of yeast extract (Fisher Chemical), and 5 g of NaCl (Fisher Chemical)/liter) supplemented with 100 μg/ml ampicillin and/or 34 μg/ml chloramphenicol where necessary. The DH5α-T1 *E. coli* strain was used for propagation of mutant plasmids, and the BL21 Codon+ (DE3) RIL *E. coli* strain (Agilent Technologies) was used for protein overexpression and purification.

The plasmid pRDK316, which is pRS316 (*URA3 ARS CEN*) encoding WT *MSH2*, was used to create the Walker A Lys mutations in *MSH2*, whereas the plasmid pRDK1227, which is pRS315 (*LEU2 ARS CEN*) encoding WT *MSH6*, was used to create Walker A Lys mutations in *MSH6* in replicating plasmids. The plasmid pRDK858, which is YIp5 (*URA*) encoding WT *MSH2*, was used to create the Walker A Lys mutations in *MSH2*, whereas the plasmid pRDK1579, which is YIp5 (*URA3*) encoding WT *MSH6*, was used to create Walker A Lys mutations in *MSH6* in integrating plasmids. The plasmid pRDK1884, which is pET11a expression vector encoding both WT *MSH2* and *MSH6*, was used to create the Walker A Lys mutations in *MSH2* and *MSH6* for protein expression. Mutant *msh2* and *msh6* mutations were created using the GeneArt Site-Directed Mutagenesis kit (Thermo Fisher), and transformations were performed using the manufacturer's protocol. The YIp5-based plasmids were used in standard pop-in pop-out protocols to replace WT *MSH2* or *MSH6* with different mutant alleles, and gene deletions were made using standard PCR-based gene deletion methods using the otherwise WT *S. cerevisiae* strain RDK5964. For genetic dominance experiments, pRS315- and pRS316-based plasmids were transformed into the otherwise WT *S. cerevisiae* strain RDK5964. All *S. cerevisiae* transformations were performed using standard lithium acetate transformation protocols. All plasmid and chromosomal mutant genes were sequenced in their entirety to confirm that only the desired mutation had been introduced. *S. cerevisiae* strains are listed in Table S1, and plasmids are listed in Table S2.

### Protein purification

*S. cerevisiae* Msh2–Msh6 (WT and mutant complexes), Mlh1–Pms1, PCNA, RFC-Δ1N, Exo1, and DNA polymerase δ were all overproduced and purified as described previously (58, 60); multiple batches of each protein were usually used during the studies described. Protein preparations were greater than 95% pure when judged using Coomassie Blue staining. The Msh2(K694M)–Msh6 and Msh2–Msh6(K988M) protein preparations were those described previously (24). Assays were optimized for each batch of protein.

### DNA substrates

The DNA substrates containing a +1 insertion mispair due to the presence of an inserted T or creation of a GT mispair in the nicked strand of the substrate were constructed following previously described methods using the mutant derivatives of pBluescript SK+, pRDK1252, and pRDK1253 (58). One of the substrates contained a single-strand break at the NaeI site 343 bp 5′ to the mispair, and the other substrate contained a single-strand break at the AflIII site 442 bp 3′ to the mispair. A ScaI site 1108 bp 5′ of the mispair was used along with the PstI site that is disrupted by the mispair to diagnose repair products in the reconstituted repair assay or to linearize the substrate for the Mlh1–Pms1 endonuclease activation assay. See Fig. 2*A* for a schematic of the substrate.

The DNA substrates used for all surface plasmon resonance experiments were 236 bp long and contained a central GT mispair or +1 (+T) insertion for mispaired substrates or a GC bp for homoduplex substrates. One end of each substrate had a conjugated biotin, whereas the opposite end encoded the *lacO* sequence. Substrates were prepared using a protocol published previously (21, 22, 40, 78).

### Mispair-directed Mlh1–Pms1 endonuclease assay

Assays were performed essentially as described (53). Briefly, the 40-μl reactions contained 73 pmol of Msh2–Msh6 (WT or mutant), 54 pmol of PCNA, 41 pmol of RFC-Δ1N, 54 pmol of Mlh1–Pms1, and 100 ng of DNA substrate and were performed in a buffer containing 20 mm HEPES, pH 7.6, 140 mm KCl, 5 mm MgCl_2_, 2 mm ATP, 1 mm DTT, 0.2 mg/ml BSA (Roche), 1.2% glycerol (v/v), and 0.5 mm MnSO_4_. After incubation at 30 °C for 30 min, reactions were stopped with addition of 30 μl of stop solution (0.35% SDS, 0.3 mg/ml Proteinase K (Sigma-Aldrich), 400 mm NaCl, 0.3 mg/ml glycogen, and 13 mm EDTA) and incubated for 15 min at 55 °C. The DNA products were purified by phenol extraction and ethanol precipitation. Purified DNA was digested with 10 units of ScaI-HF (New England Biolabs), and 10 ng were run on 1% denaturing agarose gels at 25 V for 3 h and transferred to an Amersham Biosciences Hybond-N^+^ membrane (GE Healthcare) using the capillary transfer method with 20× SSC buffer. DNA was cross-linked to the blot using the autocross-link function on a Stratagene UV Stratalinker 2400, probed with a biotinylated oligo that hybridized to the mispair-containing strand (5′-ATT ATC CCG TAT TGA CGC CGG GCA AGA GCA ACT CGG TCG CCG CAT ACA CT) for 3 h at 55 °C in UltraHyb buffer (Invitrogen), and developed using a Chemiluminescent Nucleic Acid Detection Module (Thermo Scientific). Blots were scanned on a Bio-Rad ChemiDoc MP Imaging System, quantitation was performed using Image Lab software, and graphs were made with GraphPad Prism 6.

### Reconstituted 3′ nick–directed MMR assay

Assays were performed essentially as described (58). Reactions were split into two stages. Stage 1 contained 33 mm Tris, pH 7.6, 75 mm KCl, 2.5 or 0.1 mm ATP (as indicated in the figures), 1.66 mm GSH, 8.3 mm MgCl_2_, 80 μg/ml BSA, 200 μm each dNTP, 145 fmol of PCNA, 110 fmol of RFC-Δ1N, 40 fmol of Mlh1–Pms1, 195 fmol of the indicated Msh2–Msh6 variant, and 100 ng of the 3′ AflIII DNA substrate in a 5-μl volume. Reactions were incubated for 10 min at 30 °C. The Stage 2 reaction buffer contained the same components as Stage 1 except for the addition of 1 mm MnSO_4_, and the Stage 2 proteins included 145 fmol of PCNA, 110 fmol of RFC-Δ1N, 40 fmol of DNA polymerase δ, 2.1 fmol of Exo1, 900 fmol of *S. cerevisiae* replication protein A, and 195 fmol of the indicated Msh2–Msh6 variant (note that Stage 2 did not contain Mlh1–Pms1) in a 5-μl volume. Stage 1 and Stage 2 reactions were combined and incubated at 30 °C for 2 h and then stopped by addition of 0.42 μl of 0.5 m EDTA and 20 μl of stop solution (360 μg/ml Proteinase K and 80 μg/ml glycogen) followed by incubation at 30 min at 55 °C. Reactions were phenol-chloroform–extracted, and the DNA was ethanol-precipitated followed by digestion with PstI and ScaI. Digested DNA was subjected to electrophoresis on a 0.8% agarose, 1× Tris acetate-EDTA gel for 45 min at 100 V. Gels were imaged on an Alpha Imager HP system, and quantitation was performed with AlphaView software.

### Reconstituted 5′ nick–directed MMR assay

Assays were performed essentially as described (58). The reactions contained 33 mm Tris, pH 7.6, 75 mm KCl, 2.5 or 0.1 mm ATP (indicated in the figures), 1.66 mm GSH, 8.3 mm MgCl_2_, 80 μg/ml BSA, 200 μm each dNTP, 145 fmol of PCNA, 110 fmol of RFC-Δ1N, 40 fmol of Mlh1–Pms1, 195 fmol of the indicated Msh2–Msh6 variant, and 100 ng of the 5′ NaeI DNA substrate in a 10-μl volume. Reactions were incubated for 2 h at 30 °C and stopped in the same way as the 3′ reconstituted MMR reaction. Phenol extractions, DNA precipitation, restriction endonuclease digests, and gel electrophoresis were all performed identically to the 3′ reconstituted MMR reaction.

### Surface plasmon resonance (Mlh1–Pms1 recruitment, sliding clamp formation, mispair binding, and direct dissociation)

Surface plasmon resonance was performed using a Biacore T100 instrument (GE Healthcare). All DNA substrates were 236 bp long and contained a central GT mispair, a biotin conjugated to one end, and the *lacO* sequence at the other end. For each chip, one lane was used as a blank reference, one lane contained ∼20 ng (∼100 RU) of mispaired DNA, and one contained ∼20 ng (∼100 RU) of fully base-paired DNA. The ATP concentration is indicated in the figure or 250 μm where not indicated. Where present, the ADP concentration was 250 μm, and the IPTG concentration was 1 mm. Reaction buffer contained 25 mm Tris, pH 8, 4 mm MgCl_2_, 110 mm NaCl, 0.01% Igepal, 2 mm DTT, and 2% glycerol. The assay setups were as follows: sliding clamp formation: Step 1, reaction buffer with 30 mm LacI for 60 s; Step 2, reaction buffer with 50 nm Msh2–Msh6, various concentrations of ATP, and 30 nm LacI for 150 s; and Step 3, reaction buffer with 50 nm Msh2–Msh6, various concentrations of ATP, 30 nm LacI for 150 s, and 1 mm IPTG for 90 s. Direct dissociation experiments were exactly the same except Msh2–Msh6 and IPTG were omitted from Step 3. For Mlh1–Pms1 recruitment experiments, the assays were set up as follows: Step 1, reaction buffer with 30 nm LacI for 60 s; Step 2, reaction buffer with 20 nm Msh2–Msh6, 250 μm ATP, and 30 nm LacI for 100 s; and Step 3, reaction buffer with 20 nm Msh2–Msh6, 250 μm ATP, 30 nm LacI, and 40 nm Mlh1–Pms1 for 300 s. Experiments were performed at 10 °C with a 20 μl/min flow rate, and data were collected at a frequency of 10 Hz. BiaEvaluation v3.1 and Prism 6 were used to evaluate data.

The steady-state maximum for the association curves (RU_max_) of Msh2–Msh6 binding to DNA was determined by fitting the equation RU(*t*) = RU_max_ [1 − *f*_1_ exp(−*k*_1_ (*t* − *t*_0_)) − (1 − *f*_1_) exp(−*k*_2_ (*t* − *t*_0_))] or RU(*t*) = RU_max_[1 − exp(−*k* (*t* − *t*_0_))] depending on whether the association showed biphasic or monophasic behavior. In these equations, only RU_max_ and *t*_0_, the initial time of injection, were taken to have direct physical meaning. Dissociation of Msh2–Msh6 sliding clamps from unblocked DNAs was fit to the equation RU(*t*) = *B* + (*A* − *B*)[(*f*_1_/(*f*_1_ + *f*_2_ + *f*_3_)) exp(−*k*_1_(*t* − *t*_0_)) + (*f*_2_/(*f*_1_ + *f*_2_ + *f*_3_)) exp(−*k*_2_(*t* − *t*_0_)) + (*f*_3_/(*f*_1_ + *f*_2_ + *f*_3_)) exp(−*k*_3_(*t* − *t*_0_))] where *A* and *B* corresponds to the steady-state response. Direct dissociation of Msh2–Msh6 from double end–blocked DNAs was fit to the equation RU(*t*) = *B* + (*A* − *B*)[*f*_1_ exp(−*k*_1_(*t* − *t*_0_)) + (1 − *f*_1_) exp(−*k*_2_(*t* − *t*_0_))]. The Mlh1–Pms1 recruitment experiments were fit with the equation RU(*t*) = *A*(*t*) if *t* < *t*_1_ and *A*(*t*) + *B*(*t*) if *t* ≥ *t*_1_. *A*(*t*) is the association of Msh2–Msh6, which starts at the injection start time *t*_0_; either *A*(*t*) = *A*_max_ [1 − *f*_1_ exp(−*k*_1_(*t* − *t*_0_)) − (1 − *f*_1_) exp(−*k*_2_(*t* − *t*_0_))] or *A*(*t*) = *A*_max_ [1 − exp(−*k*_1_(*t* − *t*_0_))]. *B*(*t*) is the association of Mlh1–Pms1, which starts at the injection start time *t*_1_; *B*(*t*) = *B*_max_ [1 − exp(−*k*_3_(*t* − *t*_1_))]. Molar ratios of associated Mlh1–Pms1 to Msh2–Msh6 were calculated by (*B*_max_/MW_Mlh1–Pms1_)/(*A*_max_/MW_Msh2–Msh6_) where MW_Mlh1–Pms1_ is the molecular weight of Mlh1–Pms1 and MW_Msh2–Msh6_ is the molecular weight of Msh2–Msh6.

### ATP binding assay

Assays were performed essentially as described (24) and contained differing amounts of ATP and Msh2–Msh6 for the high- and low-ATP conditions. The 20-μl reaction buffer contained 50 mm Tris, pH 8, 110 mm NaCl, 2 mm DTT, 0.1 mg/ml BSA (Roche Applied Science), 0.5 mm EDTA, and 5% glycerol (v/v). The low-ATP condition contained 0.4 pmol of Msh2–Msh6 and 1 μm ATP with a radiolabeled to unlabeled ratio of 1:6. The high-ATP condition contained 4 pmol of Msh2–Msh6 and 60 μm ATP with a radiolabeled to unlabeled ratio of 1:31. Reactions were incubated for 10 min on ice and cross-linking on ice for 20 min in a UV Stratalinker 2400 machine (approximately 5.52 J/cm^2^ total; Stratagene). Laemmli buffer was added to a 1× concentration, and the samples were incubated for 10 min at 95 °C and subjected to 4–15% SDS-PAGE for 1 h at 200 V. Gels were exposed to phosphorimaging screens and scanned on a Bio-Rad Personal Molecular Imager.

### Determination of mutation rates

Mutation rates were determined by fluctuation analysis (79) using the *hom3–10* and *lys2–10A* frameshift reversion assays and the *CAN1* forward mutation assay and were performed as described (12, 72, 80). Seven single colonies for each of two separate isolates (14 single colonies total) of the same strain were isolated from YPD plates when mutations were integrated onto the chromosome and used to inoculate 10-ml YPD cultures that were grown overnight at 30 °C with shaking. Appropriate dilutions were then plated onto YPD, synthetic complete (SC) − lysine, SC − threonine, or SC − arginine + canavanine (60 mg/liter canavanine) media. The resulting colonies were counted after growth at 30 °C for 3 days, and the median mutation rate was calculated for each strain.

Dominance of Walker A Lys mutations was assessed using semiquantitative patch tests (12, 81). *S. cerevisiae* strain RDKY5964 harboring *ARS CEN* plasmids encoding mutant versions of *msh2* or *msh6* (Table S2) were grown on −Ura (*msh2* mutations) or −Leu (*msh6* mutations) plates to obtain single colonies, which were then patched onto the same medium and after growth replica-plated onto −Ura or −Leu plates that were in addition −Thr, −Lys, or −Arg +canavanine (60 mg/liter canavanine) to test for growth of papillae. Increased papillae in a patch signified genetic dominance of the mutant allele. The *msh6-S1036P* mutation is a known dominant mutation and was used as a positive control.

## Author contributions

W. J. G. and C. D. P. investigation; W. J. G., C. D. P., and R. D. K. methodology; W. J. G., C. D. P., and R. D. K. writing-original draft; W. J. G., C. D. P., and R. D. K. writing-review and editing; C. D. P. and R. D. K. conceptualization; C. D. P. and R. D. K. formal analysis; C. D. P. and R. D. K. supervision; C. D. P. validation; R. D. K. funding acquisition; R. D. K. project administration.

## Supplementary Material

Supporting Information

## Footnotes

2 The abbreviations used are:

MMR: mismatch repair
PCNA: proliferating cell nuclear antigen
SPR: surface plasmon resonance
RU: response units
IPTG: isopropyl 1-thio-β-d-galactopyranoside
RFC: replication factor C
SC: synthetic complete
AMP-PNP: adenosine 5′-(β,γ-imino)triphosphate
MSH: MutS Homolog.
