# Supplementary material for: The properties of Msh2–Msh6 ATP binding mutants suggest a signal amplification mechanism in DNA mismatch repair
Source: J Biol Chem. 2018 Sep 20;293(47):18055–70. doi: 10.1074/jbc.RA118.005439 (PMC6254361; doi:10.1074/jbc.RA118.005439)
Supplement: Supporting Information [file supp_293_47_18055__index.html]

The properties of Msh2-Msh6 ATP binding mutants suggest a signal amplification mechanism in DNA mismatch repair — Msh2-Msh6 sliding clamps amplify mispair signals — The properties of Msh2–Msh6 ATP binding mutants suggest a signal amplification mechanism in DNA mismatch repair — Msh2–Msh6 sliding clamps amplify mispair signals — Supporting Information 

# The properties of Msh2–Msh6 ATP binding mutants suggest a signal amplification mechanism in DNA mismatch repair

## Supporting Information

- Supporting Information (to be published online) - One supporting figure and two supporting tables.
