# Supplementary material for: The properties of Msh2–Msh6 ATP binding mutants suggest a signal amplification mechanism in DNA mismatch repair
Source: J Biol Chem. 2018 Sep 20;293(47):18055–70. doi: 10.1074/jbc.RA118.005439 (PMC6254361; doi:10.1074/jbc.RA118.005439)
Supplement: Supporting Information [file supp_RA118.005439_140220_1_supp_204382_pf7rzz.pdf]

Supporting Information

The properties of Msh2-Msh6 ATP binding mutants suggest a signal amplification mechanism in DNA mismatch repair

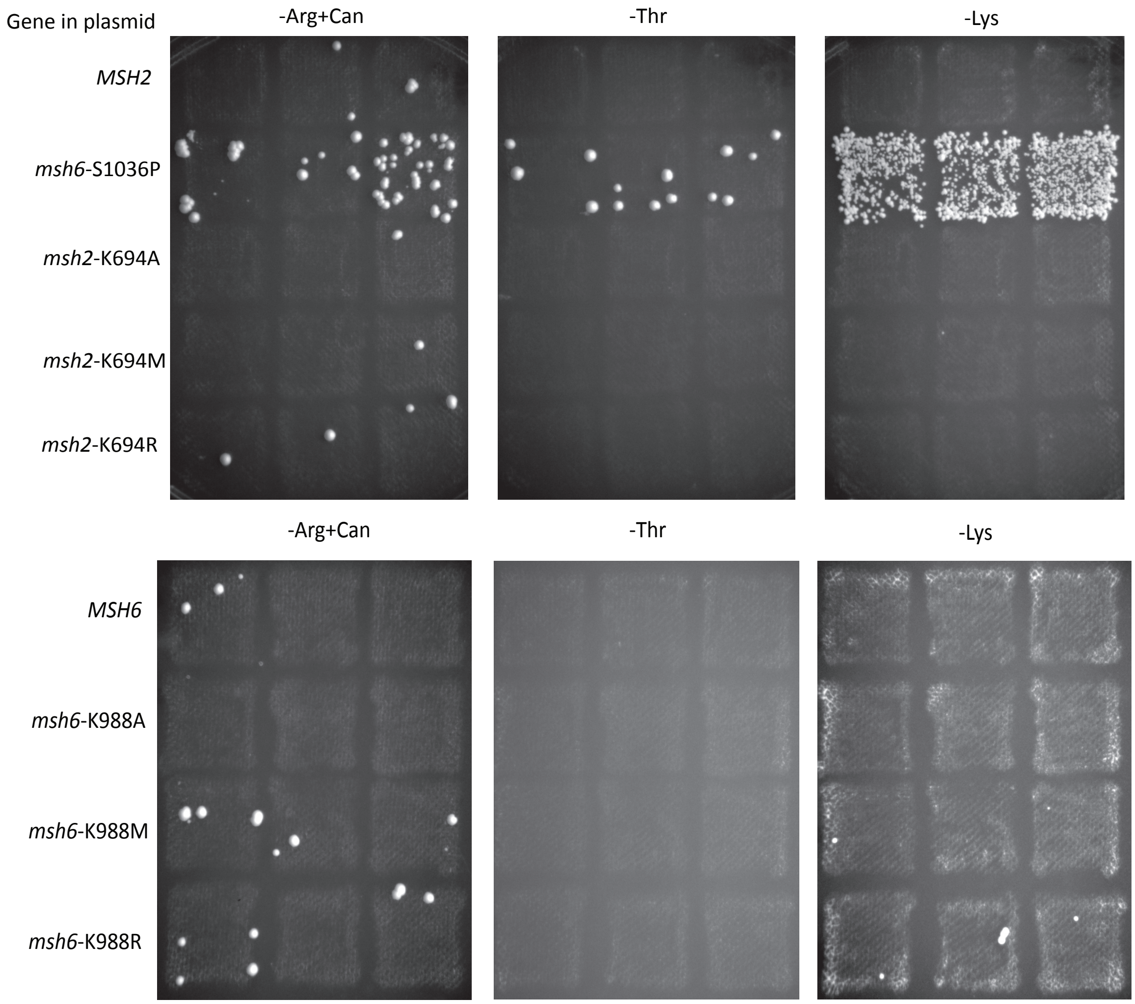

Supporting Figure S1. Triplicate patch tests showing dominance, or lack thereof, for each of the *msh2* or *msh6* mutations. A single copy plasmid containing a mutant copy of either *msh2* or *msh6* was transformed into strains encoding the *lys2-10A* and *hom3-10* frameshift reversion assays and the *CAN1* forward mutation assay. Patches of the strains were then replica plated on drop out media lacking the indicated amino acids. Increased papillae formation indicates an increased mutation rate resulting from defective MMR. Because the mutations are encoded on a plasmid and the chromosome contains a wild-type copy of the same gene, papillae formation also indicates genetic dominance of a mutation. *msh6-S1036P* is a known dominant mutation used as a positive control.

Supporting Table S1. *S. cerevisiae* Strains used in this study.

RDK Number	Genotype	Reference
5964	*MATa, ura3-52, leu2Δ1, trp1Δ63, hom3-10, his3Δ200, lys2-10A*	Reference (45)
7965	*MATa, ura3-52, leu2Δ1, trp1Δ63, hom3-10, his3Δ200, lys2-10A, msh6Δ::hphNT1*	Reference (45)
3688	*MATa, ura3-52, leu2Δ1, trp1Δ63, hom3-10, his3Δ200, lys2-10A, msh2Δ::hisG*	Reference (71)
9379	RDKY5964 *msh2-K694A*	This study
9380	RDKY5964 *msh2-K694R*	This study
9381	RDKY5964 *msh2-K694M*	This study
9382	RDKY5964 *msh6-K988A*	This study
9383	RDKY5964 *msh6-K988R*	This study
9384	RDKY5964 *msh6-K988M*	This study
9385	RDKY5964 *msh6-K988A, msh3Δ::HIS3*	This study
9386	RDKY5964 *msh6-K988M, msh3Δ::HIS3*	This study
9387	RDKY5964 *msh6-K988R, msh3Δ::HIS3*	This study

Supporting Table S2. Plasmids used in this study.

pRDK Number	Characteristics	References
1863	pET11a/ *MSH2-msh6-K988R*	This study
1864	pET11a/ *msh2-K694R-MSH6*	This study
1865	pET11a/ *MSH2-msh6-K988A*	This study
1866	pET11a/ *msh2-K694A-MSH6*	This study
1884	pET11a/ *MSH2-MSH6*	This study
361	pRS316/ *MSH2*	Reference (71)
1871	pRS316/ *msh2-K694A*	This study
1867	pRS316/ *msh2-K694M*	This study
1872	pRS316/ *msh2-K694R*	This study
1227	pRS315/ *MSH6*	Reference (71)
1868	pRS315/ *msh6-K988A*	This study
1870	pRS315/ *msh6-K988M*	This study
1869	pRS315/ *msh6-K988R*	This study
1585	pRS306/ *msh6-S1036P*	Reference (51)
1873	yIp5/ *msh2-K694A*	This study
1874	yIp5/ *msh2-K694R*	This study
1875	yIp5/ *msh2-K694M*	This study
1876	pRS306/ *msh6-K988A*	This study
1877	pRS306/ *msh6-K988R*	This study
1878	pRS306/ *msh6-K988M*	This study
